# Molecular characterization of Carbapenem resistant *Escherichia coli* recovered from a tertiary hospital in Lebanon

**DOI:** 10.1371/journal.pone.0203323

**Published:** 2018-09-06

**Authors:** Christel Dagher, Tamara Salloum, Sahar Alousi, Harout Arabaghian, George F. Araj, Sima Tokajian

**Affiliations:** 1 Department of Natural Sciences, School of Arts and Sciences, Lebanese American University, Byblos, Lebanon; 2 Department of Pathology and Laboratory Medicine, Faculty of Medicine, American University of Beirut Medical Center, Beirut, Lebanon; Leibniz-Institute DSMZ, GERMANY

## Abstract

The emergence of carbapenem resistant *Escherichia coli* represents a serious public health concern. This study investigated the resistome, virulence, plasmids content and clonality of 27 carbapenem resistant *E*. *coli* isolated from 27 hospitalized patients at the American University of Beirut Medical Center (AUBMC) in Lebanon between 2012 and 2016. Whole-genome sequencing (WGS) data were used to identify resistance determinants. Multilocus sequence typing (MLST), pulsed field gel electrophoresis (PFGE), phylogenetic grouping and PCR-based replicon typing (PBRT) were also performed. The 27 isolates were distributed into 15 STs, of which ST405 (14.8%; n = 4) was the most prevalent. All of the 27 isolates were carbapenem resistant and 20 (74%) were extended-spectrum β-lactamase (ESBL) gene carriers. The predominant detected carbapenemases were *bla*_OXA-48_ (48.1%; n = 13) and *bla*_OXA-181_ (7.4%; n = 2), for the ESBLs it was *bla*_CTX-M-15_ (55.6%; n = 15) and *bla*_CTX-M-24_ (18.5%; n = 5), and for the AmpC-type β–lactamases, *bla*_CMY-42_ (40.7%; n = 11) and *bla*_CMY-2_ (3.7%; n = 1). Thirteen replicons were identified among the 27 *E*. *coli* isolates including: IncL/M, IncFIA, IncFIB, IncFII, IncI1, and IncX3. PFGE revealed a high genetic diversity with the 27 isolates being grouped in 21 different pulsotypes. SNPs analysis and PFGE showed a possible clonal dissemination of ST405, ST1284, ST354 and ST410 and the dominance of certain STs, monitoring of which could help in elucidating routes of transmission. This study represents the first WGS-based in depth analysis of the resistomes and mobilomes of carbapenem resistant *E*. *coli* in Lebanon.

## Introduction

*Escherichia coli* are one of the most common members of the family Enterobacteriaceae and often exist as commensal in the gastrointestinal tract of humans and animals. However, they can also colonize, infect and cause both hospital- and community-acquired infections leading to serious clinical disorders at both intra- and extraintestinal sites such as urinary tract infections, septicemia, neonatal meningitis and bacteremia [1–3]. Over the past decade, there has been a worldwide increase in extended-spectrum β-lactamase (ESBL) producing and carbapenem resistant *E*. *coli* [4, 5]. A varying number of different mechanisms are thought to be involved in the resistance to carbapenems. Primarily, the process includes the production of carbapenemases like class A KPC, class B metallo-β-lactamases (IMP, VIM and NDM) as well as class D OXA-type enzymes (OXA-48-like) [6]. Carbapenem resistance may also be due to AmpC type enzymes or ESBLs along with impermeability of the membrane [7]. Membrane impermeability can be linked to modifications or absence of OmpC and/or OmpF porin channels [8] or presence of drug efflux pumps [9].

The frequency of infections caused by ESBL-producing Enterobacteriaceae increased from 2000 to 2011 in Lebanon [7]. This however, drastically surged during the period of 2012 to 2016, when Lebanon witnessed an unprecedented influx of refugees subsequently leading to the dissemination of novel multidrug resistance mechanisms of public health importance such as ESBLs and carbapenemases. In fact, of the fifteen thousand *E*. *coli* isolated from a tertiary-care center in Beirut from 2012 to 2016, 24% to 29.5% were found to be ESBL producers (personal communication). In 2009, 2% of *E*. *coli* isolates collected from the same hospital were both carbapenem resistant and ESBL-producing [10]. Since carbapenems are the last resort to treat life-threatening *E*. *coli* infections, it is important to determine and understand routes of transmission and to develop strategies to block or slow down the spread of resistant determinants. We used whole-genome sequencing (WGS) to study the characteristics and determine the genomic profiles and diversity among 27 carbapenem resistant *E*. *coli* collected from the American University of Beirut Medical Center (AUBMC) during 2012 to 2016.

## Results

### Antimicrobial resistance

**Fig 1:**
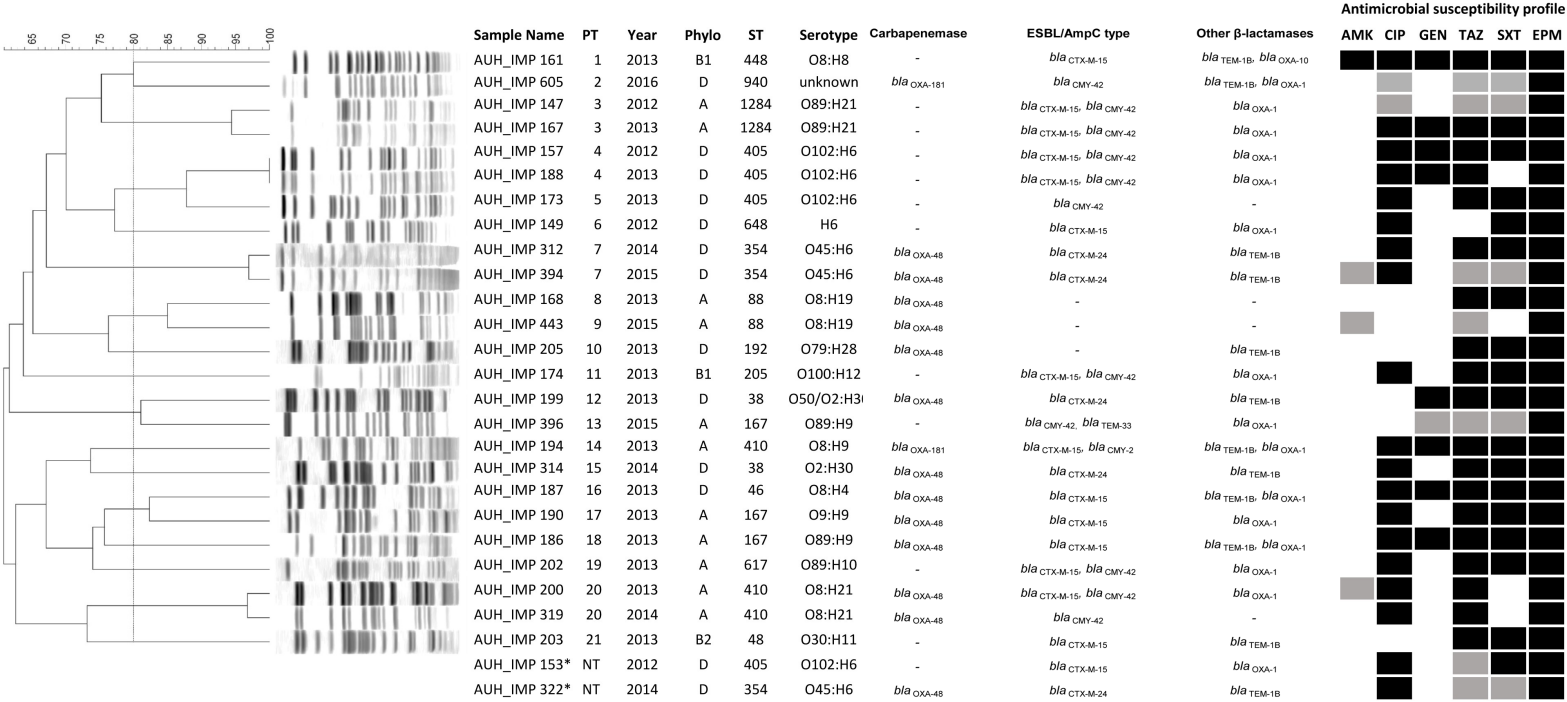
PFGE profiles, phylogenetic group, sequence types (STs), and antimicrobial susceptibility profiles of the sequenced isolates. The isolate, year of isolation, phylogroup, ST, serotype, detected carabapenemase and antimicrobial susceptibility profiles are listed after each PFGE pattern. AMK, amikacin; CIP, ciprofloxacin, ampicillin; GEN, gentamicin; TAZ, tazobactam, SXT, trimethoprim/sulfamethoxazole; EPM, ertapenem. Black indicates “resistant”, grey indicates “intermediate susceptibility” and blank indicates “sensitive”. * indicates untypable strains; NT: not typable; PT: pulsotype.

The antimicrobial resistance patterns to the tested antibiotics of the 27 *E*. *coli* isolates are summarized in Fig 1. All isolates were resistant to ertapenem, followed by 37% (n = 10) and 14.8% (n = 4) of the isolates that were resistant and intermediately resistant, respectively, to imipenem and 22.2% (n = 6) and 3.7% (n = 1) that were resistant and intermediately resistant, respectively, to meropenem. Also, 70.4% (n = 19) were resistant to ciprofloxacin and tazobactam (70.4%; n = 19), followed by trimethoprim/sulfamethoxazole (66.7%; n = 18), gentamicin (29.6%; n = 8), and amikacin (3.7%; n = 1).

**Fig 2:**
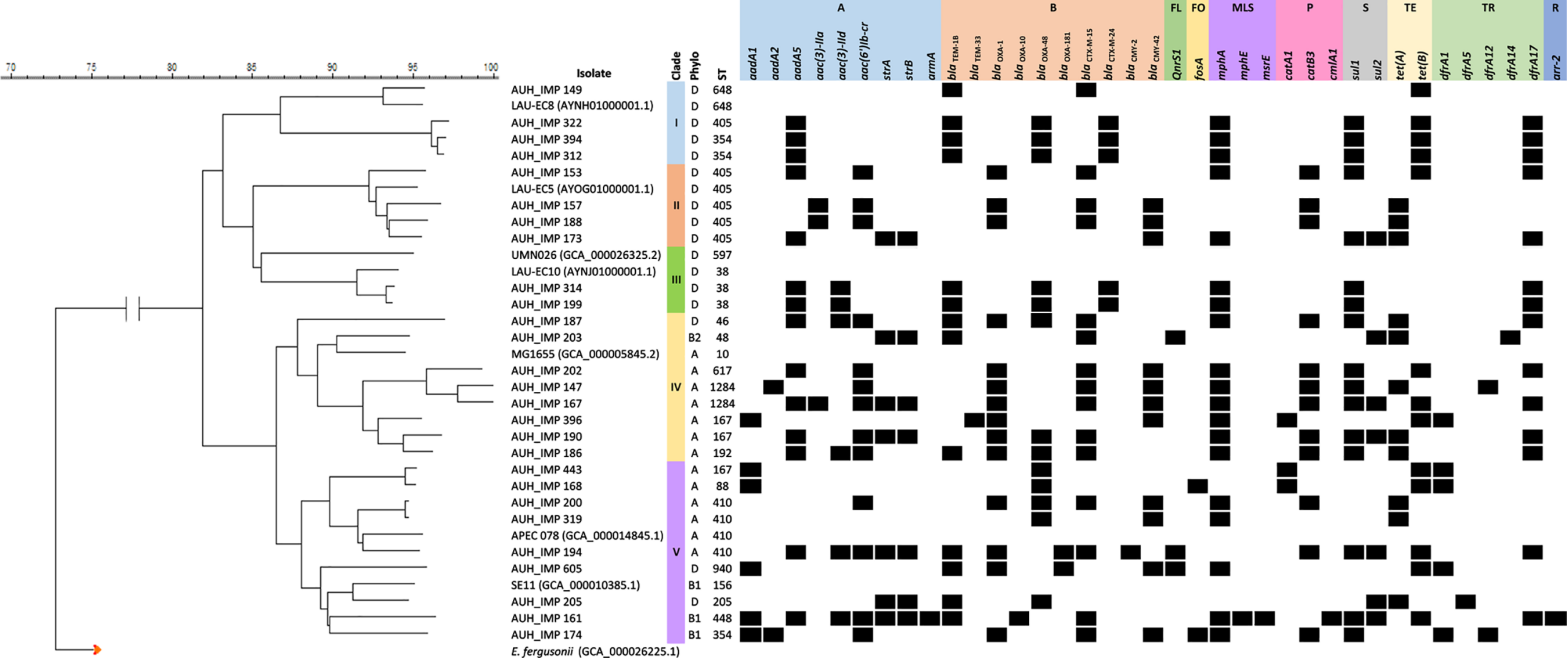
Antibiotic resistance genes detected within the draft genomes in relationship to wgSNPs distribution. Classes of antibiotic resistance genes are marked as follows: A, aminoglycoside resistance genes; B, β-lactam resistance genes; FL, fluoroquinolone resistance genes; FO, fosfomyocin resistance genes; MLS, macrolide, lincosamide and streptogramin B resistance genes; P, Phenicol resistance genes; S, sulfonamide resistance genes; TE, tetracycline resistance genes; TR, trimethoprim resistance genes and R, rifampicin resistance genes. UPGMA wgSNPs based tree was constricted using BioNumerics software version 7.6.1 (Applied Maths, Belgium).

All isolates were classified as MDR according to Magiorakos et al (2012) [11]. The isolates harbored several antibiotic resistance genes, including β-lactam and aminoglycoside resistance determinants. Among the *in silico* detected genes were: carbapenem hydrolyzing class D β-lactamase *bla*_OXA-48_ (48.1%; n = 13) and *bla*_OXA-181_ (7.4%; n = 2), extended-spectrum β-lactamases *bla*_CTX-M-15_ (55.6%; n = 15) and *bla*_CTX-M-24_ (18.5%; n = 5), other β-lactamases *bla*_OXA-1_ (51.9%; n = 14), *bla*_TEM-1b_ (48.1%; n = 13), *bla*_OXA-10_ (3.7%; n = 1), and *bla*_TEM-33_ (3.7%; n = 1), and plasmid borne AmpC cephalosporinases including *bla*_CMY-2_ (3.7%; n = 1) and *bla*_CMY-42_ (40.7%; n = 11). Aminoglycoside resistance genes *aadA5* (51.9%; n = 14) and *aac(6’)Ib-cr* (48.1%; n = 13) conferring resistance to both aminoglycoside and fluoroquinolone were also detected (Fig 2; S1 Table).

Out of the 12 isolates that were carbapenem resistant yet lacking carbapenemase encoding genes, 75% (n = 9) showed multiple deletion events and truncations in *ompC* and *ompF* porin encoding genes. Also, substitutions were observed in the OmpF and OmpC protein sequences. In addition to truncated porin proteins, isolates AUH_IMP147, AUH_IMP157, AUH_IMP167, AUM_IMP188, AUHM_IMP174 and AUH_IMP202 co-produced CTX-M-15, OXA-1 and CMY-42; Isolate AUH_IMP173 produced CMY-42; AUHM_IMP203 produced CTX-M-15 and TEM-1B; AUH_IMP396 produced TEM-33, OXA-1 and CMY-42; AUH_IMP149 produced TEM-1B, CTX-M-15 and OXA-1; AUH_IMP153 produced CTX-M-15 and OXA-1 while AUH_IMP161 produced CTX-M-15, TEM-1B and OXA-10 (S2 Table).

### Genome statistics

Paired-end libraries (Illumina) were generated from extracted DNA and fragments with sizes between 300–600 bp chosen. High-quality reads were obtained after error correction and quality trimming. The assembled genomes ranged between 75 to 342 contigs, with a G+C % content of 50.49% to 50.8%, and total reads of 4,807,211 bp to 5,394,442 bp (S3 Table).

### Plasmids

The combined results obtained from plasmid-based replicon typing (PBRT) and *in silico* replicon typing using PlasmidFinder identified the following replicons: IncA/C2 (18.5%; n = 5), IncL/M (14.8%; n = 4), IncFIA (77.8%; n = 21), IncFIB (77.8%; n = 21), IncFII (81.5%; n = 22), IncI1 (40.7%; n = 11), IncI2 (3.7%; n = 1), IncQ1 (11.1%; n = 3), IncX1 (25.9%; n = 7), IncX2 (33.3%; n = 9), IncX3 (40.7%; n = 11), and IncY (22.2%; n = 6) (S4 Table).

PLACNETw paired end reads *in silico* analysis disjointed chromosomal genomes from accessory plasmid genomes and nine of the 13 *bla*_OXA-48_ genes allocated on IncL/M plasmids were detected, while two were confirmed by PlasmidFinder, and two were missed due to Illumina short read sequencing and/or mis-assembly. The genetic environment of *bla*_OXA-48_ was assessed using both the BioNumerics software version 7.6.1 multiple genome alignment (Applied Maths, Belgium) and RAST annotation server (S1 Fig). Two copies of IS*1999*, bracketed *bla*_OXA-48_ within the composite transposon Tn*1999* in all nine isolates having *bla*_OXA-48_ present on IncL/M.

Of importance, two IncX3 plasmids carried a *bla*_OXA-181_ carbapenemase gene (AUH_IMP194 and 605). AUH_IMP605 was characterized by Bitar et al. (2018) [12]. Moreover, IncI1 plasmids carrying the *bla*_CMY-42_ showed the presence of IS*Ecp1* insertion sequence upstream of *bla*_CMY-42_ gene in the eleven CMY-42 positive isolates (40.7%; n = 11).

### Phylogenetic grouping, MLST and serotyping

*In silico* analysis of the 27 sequenced carbapenem resistant *E*. *coli* was performed and their phylogroups, STs, and serotypes were determined. Isolates had the following distribution: A (33.3%; n = 9), B1 (7.4%; n = 2), B2 (11.1%; n = 3) and D (48.1%; n = 13). A total of 15 different STs were detected among the studied isolates (Fig 1). The most frequent ST was ST405 (14.8%; n = 4) of serotype O102:H6. Other detected STs and serotypes included: ST410 (11.1%; n = 3) of serotype O8:H21, ST354 (11.1%; n = 3) of serotype O45:H6, ST167 (11.1%; n = 3) of serotype O9:H9 (3.7%; n = 1) and O89:H9 (7.4%; n = 2), ST38 (7.4%; n = 2) of serotype O2:H30, ST88 (7.4%; n = 2) of serotype O8:H19, ST1284 (7.4%; n = 2) of serotype O89:H21, ST46 (3.7%; n = 1) of serotype O8:H4, ST48 (3.7%; n = 1) of serotype O30:H11, ST192 (3.7%; n = 1) of serotype O79:H28, ST205 (3.7%; n = 1) of serotype O100:H12, ST448 (3.7%; n = 1) of serotype O8:H8, ST617 (3.7%; n = 1) of serotype O89:H10, ST648 (3.7%; n = 1) of serotype H6 and one untypable ST940.

### Virulence determinants

**Fig 3:**
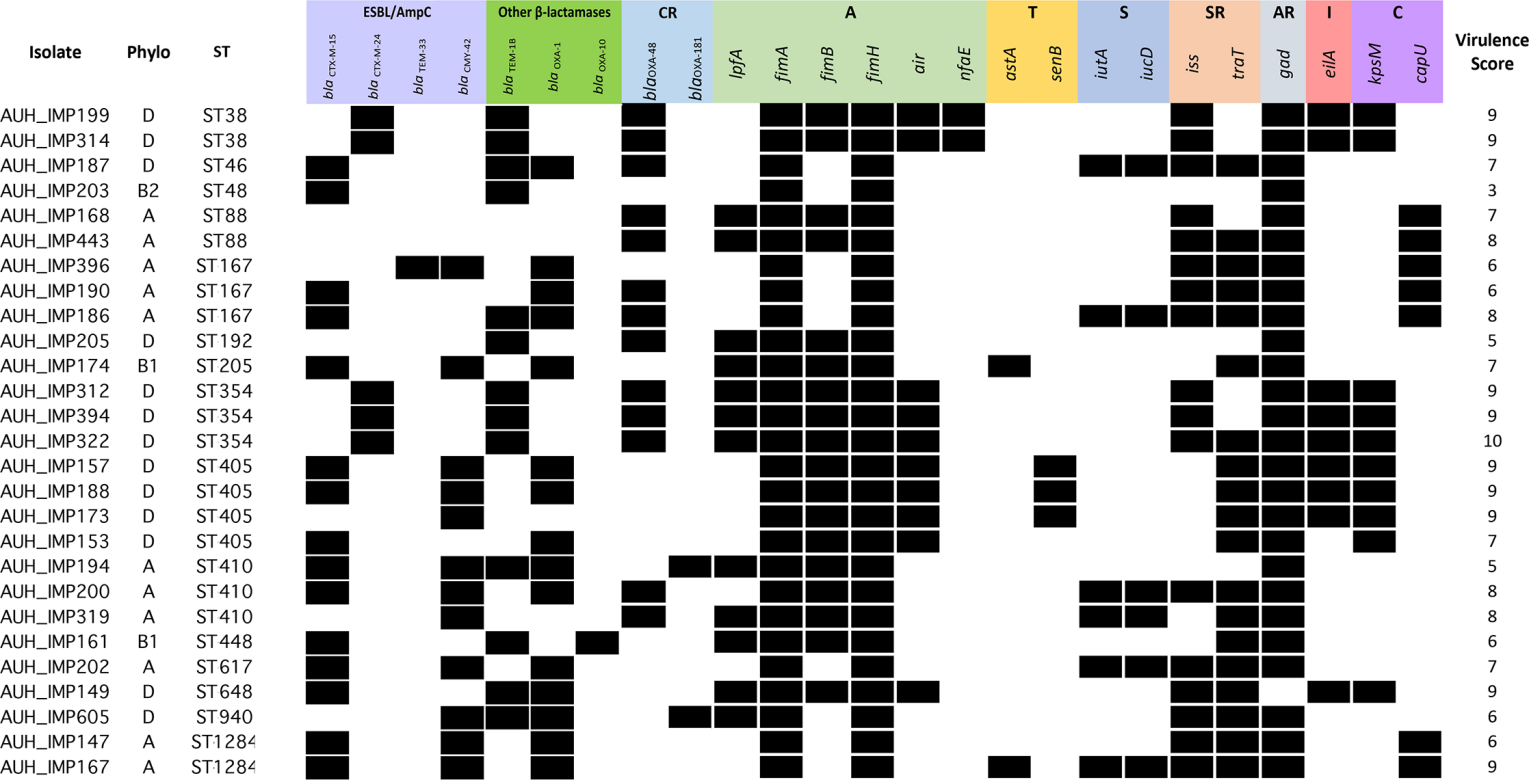
Virulence determinants detected in the 27 *E*. *coli* isolates undertaken in this study. Virulence genes we marked as follows: A, adhesins; T, toxins; S, siderophores; SR, serum resistance; AR, acid resistance; I, invasion genes and C, capsular genes. VF# is the number of virulence genes that were detected in an isolate; Phylo: phylogroup; ST: sequence type; CR: carabapenemase encoding genes.

Putative virulence factors (VFs) were identified using the CGE VirulenceFinder 1.5 tool (Fig 3). The aerobactin siderophore-encoding gene clusters *iutA* and *iucD* were detected in 22.2% (n = 6) of the isolates including *bla*_OXA-48_ positive isolates belonging to ST46, ST167 and ST410 and in *bla*_OXA-48_ negative isolates belonging to ST617 and ST1284. *gad* was detected in all the isolates except AUH_IMP149 (96.3%; n = 26). Genes encoding adhesins (*lpfa*, *fimA*, *fimB*, *fimH*, *air* or *nfae*) were also found in all the isolates, in addition to toxins, such as *senB*, that was identified in three *bla*_OXA-48_ negative ST405 isolates (11.1%), *astA* in *bla*_OXA-48_ negative ST205 and ST1284 isolates (7.4%; n = 2). Additionally, *iss* encoding an outer membrane lipoprotein that enhances serum resistance was detected in 63% (n = 17) of the isolates of various STs. The capsular gene, *kpsM*, was detected in *bla*_OXA-48_ positive ST38 isolates (7.4%; n = 2), ST405 (14.8%; n = 4) and *bla*_OXA-48_ negative ST648 (3.7%; n = 1). The highest occurrence of VFs was detected in one isolate belonging to phylogroup D and ST354 (AUH_IMP322).

### PFGE and wgSNPs phylogenetic analysis

The PFGE profiles of the *E*. *coli* isolates were analyzed. Using an 80% similarity cutoff point revealed that most of the isolates had a unique PFGE profile clustering into 21 distinct pulsotypes (Fig 1). wgSNPs-based phylogenetic typing separated the isolates based on their STs and phylogroups in accordance with the used references into five distinct clades (Fig 2). The obtained results suggested a possible clonal transmission of certain STs. For instance, all four ST405 phylogroup D isolates, clustered together having slightly different resistance profiles. Similarly, the three ST410 isolates belonged to phylogroup A clustered together with two of these isolates being PT-20 and collected in 2013 and 2014. In line with this, the two ST1284 isolates were 98% identical based on wgSNPs, had the same pulsotype (PT-3), and were collected consecutively in 2012 and 2013. Despite the association between the STs and wgSNPs based clustering of some of the isolates, we were not able to further investigate and deduce clonal relatedness and transmission. This was mainly attributed to the lack of a full clinical history, which was the major limitation and shortcoming of the study.

## Discussion

In this study, the genomic characteristics of carbapenem resistant clinical *E*. *coli* isolates collected from a tertiary hospital in Lebanon were investigated by determining and correlating their resistance gene content, STs, serotypes, plasmid content and replicon types, and VFs.

The increase in ESBL and CRE is a trend observed in hospital settings worldwide and in Lebanon. In 2003, 2.0% of *E*. *coli* recovered from Lebanon were reported as being ESBL producers [13]. An increase in ESBL-producing *E*. *coli* from 2.3% in 2000 to 16.8% in 2009 was later reported by Daoud et al. (2011) [14], and from 4% in 2000 to 30% in 2011 [7]. Similarly, the rates of carbapenem resistant Enterobacteriaceae increased from 0.4% between 2008–2010 to 1.6% in 2012 and were predominantly associated with OXA-48-producing *E*. *coli* [15, 16]. A general surveillance of carbapenem resistant Enterobacteriaceae performed during 2008 to 2014 in Lebanon, showed that OXA-48 was the most prevalent carbapenemase in the country with 91% of the isolates harboring the *bla*_OXA-48_ gene [17], with it being 73% in *E*. *coli* specifically [15]. This was in accordance with our results where carbapenemase resistance was linked to OXA-48 production in (48.1%; n = 13) of the isolates.

The isolates in this study harbored a plethora of resistance genes, two being positive (AUH_IMP194, and 605) for *bla*_OXA-181_ carbapenemase gene carried on an IncX3 plasmid as detailed by Bitar et al. (2018) [12]. *bla*_CMY-2_ (n = 1) and *bla*_CMY-42_ (n = 11) were also detected in 44.4% (n = 12) of the isolates. *bla*_CMY-2_ in general_,_ has a broad geographic distribution and is the most prevalent plasmid-mediated AmpC β-lactamase worldwide [18, 19], but was not as prevalent in Lebanon as shown by the findings of this study. The *bla*_CMY-42_ gene was detected on IncI1 plasmids, which was in harmony with Ingti et al. (2018) that reported the propagation of *bla*_CMY-42_ on IncI1-type plasmids in *E*. *coli* ST5377, ST361 and ST672 collected in India between 2012 and 2013 [20]. Studying its genetic environment, we observed upstream of the *bla*_CMY-42_ the two genes previously reported by Singh et al. (2018) [21]: *blc* and *sugE* encoding for an outer membrane lipoprotein and a drug efflux pump, respectively. We additionally detected an intact IS*Ecp1* upstream of *bla*_CMY-42_, which was not as previously shown by Hentschke et al. (2011) [22] disrupted by IS*1*.

Isolates harboring *bla*_OXA-48_ (48.1%; n = 13) in this study were resistant to ertapenem. *bla*_OXA-48_ was located on the IncL/M plasmid, which is a common *bla*_OXA-48_ finding [23]. These plasmids usually have comparable sizes, they are self-conjugative, and do not carry any other resistance determinants [24, 25]. OXA-48 was first identified in *K*. *pneumonia*e isolated from Turkey in 2001 [26] whereas in 2016, 92% of carbapenemase-producing Enterobacteriaceae in Turkey were found to be OXA-48-like producers [27] compared to other countries such as Morocco where only 5.42% of isolates collected in 2011 were OXA-48 positive [23].

CTX-M-15 was detected in 15 (55.6%) of the sequenced isolates but was not the only detected ESBL-type. CTX-M-24 was seen in five isolates (18.5%), with none being additionally positive for CTX-M-15. ST354 and ST38 strains, previously reported as being positive for CTX-M-24 and CTX-M-9, colonized canine gastrointestinal tracts, and were linked to extraintestinal infections in dogs and humans [28]. The three ST354 isolates (AUH_IMP312, 322 and 394) in this study were similarly positive for CTX-M-24, and all were ciprofloxacin resistant. These findings shed the light on the zooanthroponotic nature of such isolates with a clear evidence on the possible bi-directional movement of MDR strains between humans and pets.

Among the detected plasmids is the IncF, which has a narrow host-range and the majority of virulence-associated plasmids in *E*. *coli* were found to belong to the F incompatibility group [29]. IncF plasmids frequently harbor *bla*_CTX-M-15_ and are often associated with *bla*_TEM-1_, *bla*_OXA-1_, and *aac(6*′*)-Ib-cr* [29]. Moreover, plasmid pCoo, which was detected in three of our isolates (AUH_IMP186, 187, and 443), was the first CF-encoding plasmid detected in Enterotoxigenic *E*. *coli* (ETEC). Sequencing revealed that it contains regions homologous with plasmid R100 from *Shigella* spp. [30, 31], and carries genes encoding for virulence determinants such as CS1 fimbriae, polysaccharide deacetylase, and Ser-protease associated with CS1 fimbriae [32]. Finally, IncX3 plasmid, a narrow-host-range plasmid of Enterobacteriaceae, was detected in 10 of the studied isolates. IncX3 carried type IV secretion proteins in AUH_IMP194, 205 and 605 and *bla*_TEM-1B_ in the latter and in AUH_IMP314, and this was in harmony with previous reports [33].

Multireplicon plasmids with different FAB formulas were common. Ten FAB formulas were seen in 14 isolates (51.9%) with the most common being F1:A1:B16, detected in three isolates of phylogroup D and MLST sequence type ST405. Interestingly, the second most common FAB formula was F31:A4:B1 found in isolates of phylogroup A and sequence type ST410, while a third ST410 isolate carried a plasmid with a FAB formula F1:A1:B49. The multireplicon nature of these plasmids implicates a broader host range [32].

The observation that all isolates in this study were carbapenem resistant and yet only few carried carbapenemase genes is attributed to one of the several factors including: alterations of penicillin binding proteins due to mutations and/or reduction of outer membrane permeability associated with porin loss, and/or the overexpression of an ESBL or an AmpC enzyme [34]. Moreover, previous studies have shown that the overexpression of CTX-M-15 along with decreased membrane permeability could also lead to an increase in carbapenem resistance [35]. On the other hand, 51.8% (n = 14) and 48.1% (n = 13) of the isolates carried *bla*_OXA-1_ and *bla*_TEM-1_ respectively, the overexpression of which when combined with the loss of OmpF and OmpC porins could also lead to a higher catalytic activity towards carbapenems [34]. It’s noteworthy, that the *bla*_CMY-42_ found in 40.7% (n = 11) of the isolates encodes resistance to third generation cephalosporins, and along with loss of porins results in carbapenem resistance [36].

Different MLST types were detected in this study. ST405, which was the most common, was associated with the worldwide spread of *bla*_CTX-M-15_ and *acc(6’)-Ib-cr* [37]. All ST405 isolates except for AUH_IMP173, harbored both genes. ST405 has a wide global distribution formerly detected in the Unites States [38], Japan [39], Norway [40], and also Lebanon [41]. Furthermore, ST410 was another important ST in this study, and was previously reported in countries such as Egypt and Germany, and a common circulating clone within humans, animals, and water sources [42–44].

*E*. *coli* phylogroups reflect functional and evolutionary differences with phylogroup B2 being ancestral and A and B being sister clades [45, 46]. All phylogroups, as observed in this study, exhibit considerable genetic heterogeneity, especially group D [47]. wgSNPs-based phylogenetic analysis was also in accordance with the isolates’ phylogroups. Two ST88 (AUH_IMP168, and 443) and three ST410 (AUH_IMP200, 319, and 194) type isolates were shown to be closely related using wgSNP phylogenetic analysis. This was in agreement with the formerly published comparative analysis by Falgenhauer et al. (2016) highlighting MLST differences in only a single locus (*purA*) between the two STs [48].

The findings of this study provided an insight on the mobilome of carbapenem resistant *E*. *coli*, and highlighted on the degree of strain heterogeneity. The large diversity of plasmid-encoded resistance genes in different *E*. *coli* serotypes was an important observation, the understanding of which can help in limiting the transmission of drug-resistant determinants within and across health care institutions. More in depth studies on the clonal dissemination of MDR *E*. *coli* are highly recommended to better understand its routes of transmission.

## Materials and methods

### Ethical approval

Ethical approval was not required as clinical isolates were collected and stored as part of routine clinical care. Clinical isolates and patient records/information were anonymous and de-identified prior to analysis

### Clinical setting and bacterial isolates

*E*. *coli* isolates were collected and screened for carbapenem resistance using ertapenem disks at the Clinical and Microbiology Laboratory at AUBMC between 2012 and 2016 following Clinical and Laboratory Standards Institute (CLSI) guidelines [49]. AUBMC is one of the largest tertiary-care centers in Lebanon. It provides tertiary services for over 300,000 patients annually with a 350-bed inpatient capacity, occupied by an expatriate population from all over Lebanon as well as neighboring countries.

### Bacterial identification and susceptibility testing

Isolates were identified to species level using API20E kits (bioMérieux, Marcy l’Étoile, France) and 16S rRNA sequencing. All identified *E*. *coli* isolates were tested for resistance to amikacin, ciprofloxacin, gentamicin, tazobactam, trimethoprim/sulfamethoxazole and ertapenem (Biorad, Hercules, CA) by the disk agar diffusion technique. The zone diameters of each drug were interpreted using the criteria published by the CLSI [49].Isolates, which showed intermediate resistance, or resistance to ertapenem were further tested for confirmation by MIC determination using Etest strips for ertapenem, meropenem, and imipenem. All these isolates (n = 27) were further characterized using WGS.

### PCR screening

Bacterial DNA was extracted using the NucleoSpin® Tissue kit (Macherey-Nagel, Germany) following manufacturer’s instructions.

The phylogenetic origin of all isolates was determined by triplex PCR as previously reported [50].

Plasmid identification was performed using the DIATHEVA PBRT kit (Diatheva, Fano, Italy). Twenty-eight reference plasmids supplied by the kit were used as positive controls and included for all performed reactions [29]. All PCR reactions were performed according to the manufacturers’ instructions and visualized on a 2.5% agarose gel stained with ethidium bromide.

### PFGE

PFGE fingerprinting was performed using the XbaI restriction enzyme (ThermoScientific, Waltham, MA, USA), 1% SeaKem agarose gel and the universal laboratory standard *Salmonella enterica subsp*. *enterica serovar Braenderup* (ATCC® BAA664™) according to the standard PulseNet protocol (http://www.pulsenetinternational.org). Electrophoresis was performed using the Bio-Rad laboratories CHEF DR-III system (Bio-Rad Laboratories, Bio-Rad Laboratories Inc., Hercules, CA, USA) under the conditions set for non-O157 *E*. *coli* strains (https://www.cdc.gov/pulsenet/). Gels were stained with ethidium bromide. For samples showing identical pulsotypes or were untypable by XbaI, PFGE was repeated using the secondary enzyme AvrII (ThermoScientific, Waltham, MA, USA). PFGE profiles were analyzed with the BioNumerics software version 7.6.1 (Applied Maths, Belgium), with profiles assigned as different pulsotypes if three or more bands were different between the two of them. Pulsotypes were clustered based on the BioNumerics software analysis through dice correlation coefficients with an optimization of 1% and tolerance of 1%.

### Whole-genome sequencing

Genomic DNA (gDNA) was used as input for library preparation using the Illumina Nextera XT DNA library preparation kit (Illumina, San Diego, CA, USA). The kit was used to simultaneously fragment and tag the library, as per the manufacturer’s instruction. The library was normalized by bead-based affinity and then sequenced using the MiSeq version 3 600-cycle kit (Illumina) to perform 300 bp paired-end sequencing on the MiSeq instrument (Illumina), according to the manufacturer’s instructions. The assembly of the genomes was performed *de novo* using A5 with the default parameters [51], and the assembled draft genomes were the subjected to annotation using RAST [52].

### *In silico* analyses

The Achtman Multilocus sequence typing (MLST) and pMLST typing were performed on all isolates using the MLST web server MLST 1.8 and pMLST 1.4 respectively (www.genomicepidemiology.org) [53, 54]. Identification of virulence genes was performed using VirulenceFinder 1.5 and identification of serotypes was established using SerotypeFinder 1.1 [55]. Antibiotic resistance genes in the genome assemblies were identified by ResFinder 2.1 and plasmid typing was achieved utilizing PlasmidFinder 1.3 [56]. Phage identification was performed using the publically available Phage Search Tool (PHAST) (http://phast.wishartlab.com/index.html) [57].

PLACNETw was used to reconstruct plasmids from raw reads [58]. Genome alignments and comparisons were performed using BioNumerics software version 7.6.1 (Applied Maths, Belgium). Outer membrane porin proteins, OmpF and OmpC, were examined *in silico* using BioNumerics software in all the isolates that were negative for carbapenemase encoding genes using intact *E*. *coli* K-12 substr. MG1655 (accession # NC_000913.3) *ompC* and *ompF* genes as references, respectively.

## Supporting information

S1 Fig Comparative analysis of the genetic environments of *bla*_OXA-48_. Alignment was performed against Kp11978 plasmid pOXA-48 (Accession # JN626286.1); Two copies of IS*1999* bracketed *bla*_OXA-48_ making the composite transposon Tn*1999*; Green lines indicate inverted aligned sequences.
(TIF)

S1 Table Combined samples information and obtained results of the 27 sequenced *E*. *coli* isolates. F: female; M: male; A, adhesins; T, toxins; S, siderophores; PT: pulsotype; Phylo: phylogroup.
(XLSX)

S2 Table Analysis of the *ompC* and *ompF* in the 27 *E*. *coli* isolates. Nt: nucleotides sequence; AA: amino acids sequence; Δ: deletion.
(XLSX)

S3 Table Characteristics of the sequenced genomes. (XLSX)

S4 Table Distribution of replicons identified among the 27 *E*. *coli* isolates by using the PlasmidFinder 1.3 Web server and PBRT replicon typing. (XLSX)
