# Supplementary figures and images for: Molecular characterization of Carbapenem resistant Escherichia coli recovered from a tertiary hospital in Lebanon
Source: PLoS One. 2018 Sep 6;13(9):e0203323. doi: 10.1371/journal.pone.0203323 (PMC6126819; doi:10.1371/journal.pone.0203323)

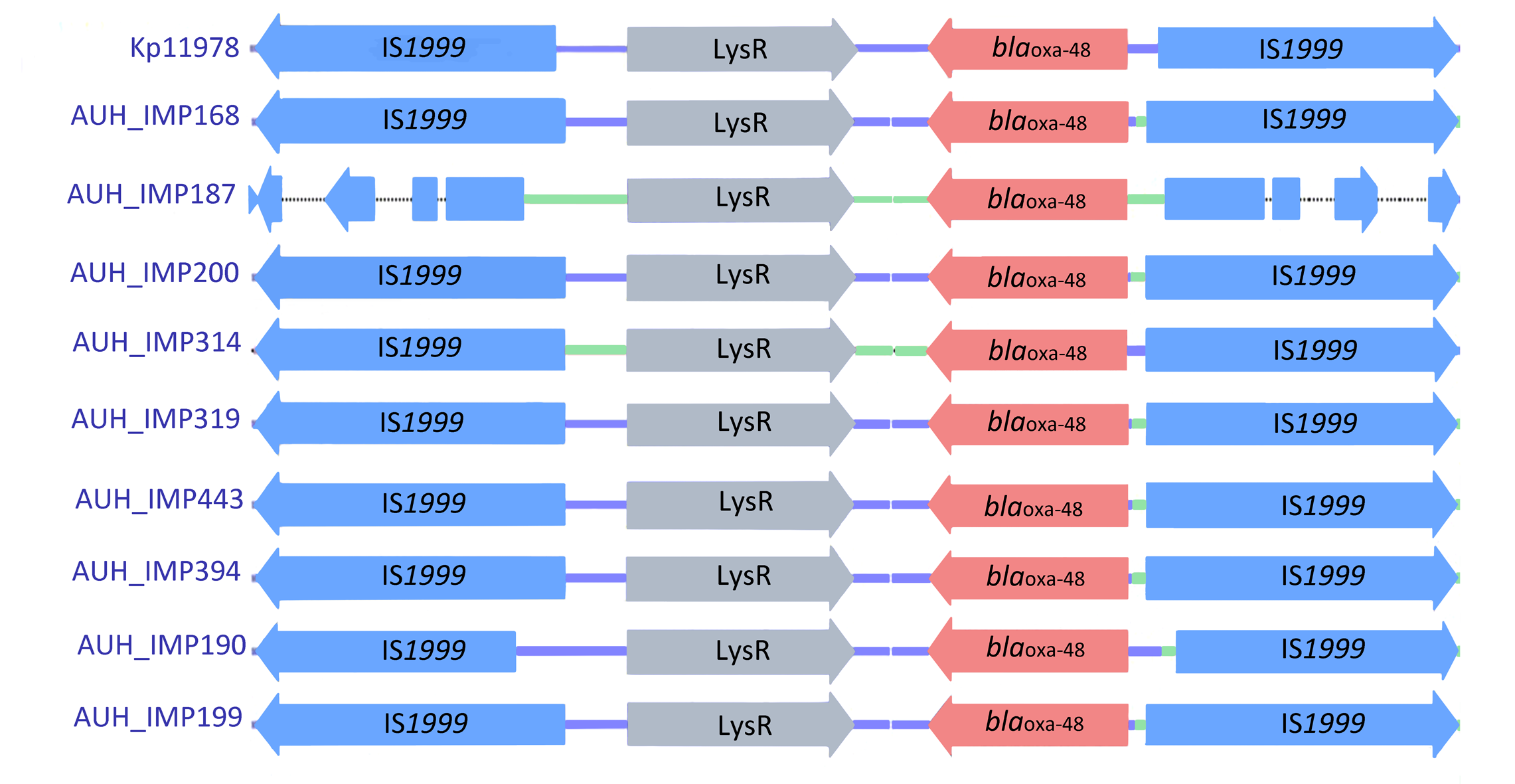

Supplement: S1 Fig — Alignment was performed against Kp11978 plasmid pOXA-48 (Accession # JN626286.1); Two copies of IS1999 bracketed blaOXA-48 making the composite transposon Tn1999; Green lines indicate inverted aligned sequences. (TIF) [file pone.0203323.s001.tif]
